# Use of dupilumab in eczematous eruptions following hematopoietic cell transplantation

**DOI:** 10.1016/j.jdcr.2025.06.060

**Published:** 2025-08-16

**Authors:** Jenna K. Dick, Andrew Truong, Christina Boull, Daniel D. Miller, Sheilagh M. Maguiness, Christen Ebens

**Affiliations:** a Division of Pediatric Dermatology, Department of Dermatology, University of Minnesota, Minneapolis, Minnesota; b Division of Dermatopathology, Department of Dermatology, University of Minnesota, Minneapolis, Minnesota; c Division of Blood and Marrow Transplant & Cellular Therapy, Department of Pediatrics, University of Minnesota, Minneapolis, Minnesota

**Keywords:** atopic dermatitis, dupilumab, GVHD, hematopoietic cell transplantation

## Introduction

Patients can experience a wide variety of inflammatory skin disorders after allogeneic hematopoietic cell transplant (HCT). The pathogenesis is complex and multifactorial involving chemo- or radiotherapy induced tissue damage, alloreactive immune responses, immune system dysregulation, immunosuppressive medication use, and impaired skin barrier function. Eczematous dermatitis has been reported in patients after HCT. Acquired eczematous dermatitis has been documented in patients without a personal or family history of atopy after transplant.^1^^,^^2^ It can present as either de novo eczematous dermatitis, potential transfer of atopic predisposition from donor to recipient, or a subtype of graft-versus-host disease (GVHD).^3^^,^^4^ A distinct variant of cutaneous GVHD, known as atopic dermatitis (AD)-GVHD is a type of eczematous dermatitis that mimics AD and has a more favorable outcome compared with other GVHD subtypes.^3^ In AD-like GVHD, the main clinical features include pruritus, xerosis, dermatitis, scaling, and perifollicular accentuation.^5^^,^^6^ Increased in circulating eosinophils and IgE have also been described in AD-GVHD.^6^ AD-like GVHD was first described in 2013 as a subset of chronic cutaneous GVHD, however it has more recently been applied in cases of acute GVHD.^3^

Eczematous dermatitis post-HCT, both acute and chronic, often requires both systemic immunotherapy and topical treatments; it can be challenging to treat with conventional therapies. Recently, dupilumab has been reported to be effective in the treatment of AD-like GVHD in pediatric and adult populations.^1^^,^^2^^,^^7^^,^^8^ Dupilumab is a human monoclonal antibody against interleukin-4 receptor alpha (IL-4Rα) that blocks IL-4 and IL-13 production and is US Food and Drug Administration–approved for treatment of AD in patients ages 6 months and older.^9^^,^^10^ Here, we report 3 patients who experienced eczematous dermatitis after HCT and were refractory to first- and second-line GVHD treatments who had observed clinical and symptomatic improvement after treatment with a short course of dupilumab.

## Case series

### Case 1

Our first patient is a 15-year-old boy with autosomal dominant STAT3 deficiency (*STAT3* c.1909G>A, p.V637M) causing Hyper-IgE syndrome who underwent human leukocyte antigen (HLA)-matched sibling donor HCT for severe manifestations including recurrent sinopulmonary infections, chronic lung disease with bronchiectasis, allergic rhinitis, asthma, eosinophilic esophagitis, and AD. The donor had no history of atopy. He was treated with dupilumab with a loading dose of 400 mg and then 200 mg every 2 weeks for 2 years until his transplant; it was discontinued 2 days before starting his chemotherapy regimen. He had no symptoms of AD in the months leading up to his transplant. His risk factors for GVHD include a history of AD and exposure to myeloablative conditioning. GVHD prophylaxis included posttransplant cyclophosphamide 50 mg/kg on days +3 and +4 after HCT, mycophenolate mofetil from day +5 to +35, and tacrolimus from day +5 (with plan to start taper at day +180 in the absence of GVHD).

**Fig 1:**
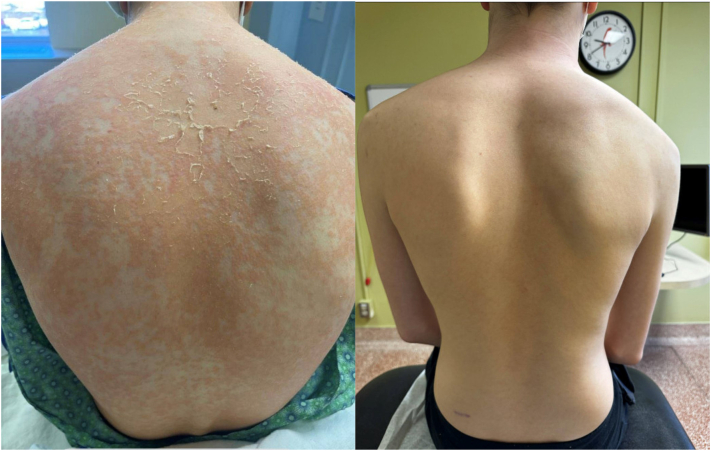
Initial presentation of eczematous dermatitis in acute graft-versus-host disease and 1 month after initial loading dose and maintenance dose of dupilumab.

On day +98 on therapeutic tacrolimus, he experienced a pruritic, papular eruption on his head, neck, trunk, and extremities (Fig 1). A skin biopsy was performed and was consistent with “acute GVHD grade II/V.” The pathology reports describe mixed features of spongiotic and interface dermatitis of the vacuolar type with some hydropic basal cell degeneration. It is noted that there is chronic lymphocytic inflammatory infiltrate with a few eosinophils. There were no hepatic or gastrointestinal GVHD manifestations. He was initially treated with topical triamcinolone 0.1% ointment to the body and hydrocortisone 2.5% cream to the face. With no improvement in the rash, sirolimus was added to tacrolimus for oral systemic immune suppression 3 days later. Despite this, over the next 2 days his rash rapidly progressed to exceed 90% body surface area (stage 3). Sirolimus was discontinued in favor of oral prednisone (2 mg/kg/d). However, the patient did not substantially improve on oral prednisone alone. Given his clinical history, dupilumab was restarted with a loading dose of 400 mg and then 200 mg every 14 days. His cutaneous findings dramatically improved over subsequent weeks, and he had sustained improvement while systemic steroids were tapered. He noted no side effects from the dupilumab (Fig 1). Dupilumab was discontinued after 7 months, and the patient’s skin has remained clear.

### Case 2

**Fig 2:**
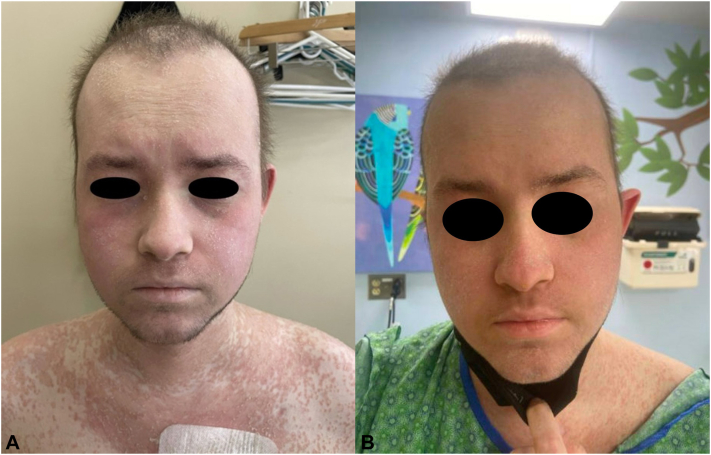
A patient with eczematous dermatitis in overlap graft-versus-host disease. **A,** Physical examination demonstrating coalescing erythematous papules into larger plaques on head and upper portion of the chest with mild-to-moderate scaling of the eyes, scalp, and cheeks. **B,** After 2 months of dupilumab.

**Fig 3:**
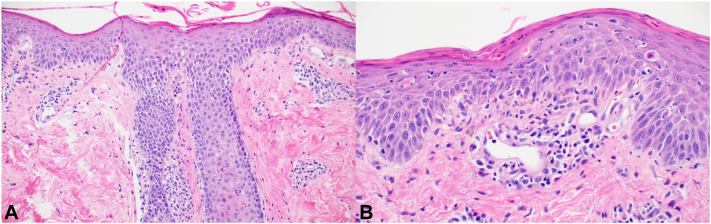
Histopathologic digital slides and descriptions from the lower portion of the left forearm. **A,** Epidermal and follicular spongiosis, with follicular necrotic keratinocytes, the latter characteristic of graft-versus-host disease GVHD. **B,** Mixed features of spongiotic and interface dermatitis, with necrotic keratinocytes at and above the basal layer. (**A** and **B,** Hematoxylin-eosin stain; original magnifications: **A,** ×100; **B,** ×200.)

A 19-year-old man underwent 5 of 6 HLA-matched umbilical cord blood transplant for acute myeloid leukemia. The donor and patient had no previous history of atopy. His risk factors for GVHD included a mismatched donor source and exposure to myeloablative conditioning. GVHD prophylaxis included mycophenolate mofetil from day −3 to +30 and tacrolimus from day −3 (with plan to start taper at day +100 in the absence of GVHD). His transplant course was complicated by acute upper gastrointestinal and then later cutaneous GVHD beginning 3 months after transplantation while on therapeutic tacrolimus (overlap GVHD). The cutaneous GVHD was describe as a morbilliform eruption involving the back, chest, arms, and legs (Fig 2). Both organ sites involved were biopsy-proven. The biopsy described, epidermal and follicular spongiosis, with follicular necrotic keratinocytes, the latter characteristic of GVHD (Fig 3, *A, B*). Escalation of immunosuppressive therapies were then added, including enteral budesonide and beclomethasone, with sequential addition of oral sirolimus then prednisone (1 mg/kg/d). This regimen rapidly improved his gastrointestinal disease (peak stage 3 with associated 15% weight loss), but his cutaneous disease continued to progress. Topical triamcinolone 0.1% ointment with wet wraps was added unsuccessfully.

The patient had increasing cutaneous involvement at serial follow-up visits, eventually exceeding >90% of his body surface area (stage 3). With severe overlap/chronic GVHD, ruxolitinib was added into his regimen and prednisone increased to 2 mg/kg/d. Weekly rituximab infusions were added 1 month later owing to elevated CD19 count and progression. Despite these treatments, he reported severe pruritus associated with his rash that was significantly impacting his overall quality of life. His pruritus did not respond to diphenhydramine, lorazepam, hydroxyzine, or aprepitant. Dupilumab was therefore added to address his symptoms and to treat any underlying eczematous component of his GVHD. He was started on dosing of 300 mg every 14 days without a loading dose. Two months after initiation, he reported the resolution of pruritus and significant improvement in erythema (see Fig 2). He noted no side effects while on the dupilumab. Unfortunately, 1 month later, the patient passed away after developing an *Aspergillus* pneumonia.

### Case 3

**Fig 4:**
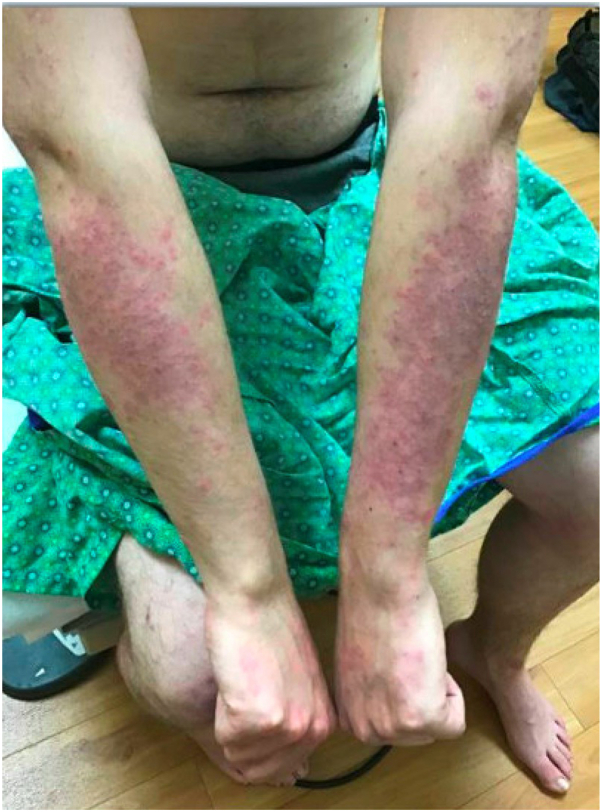
A patient with psoriatic-autoimmune reaction. Physical examination demonstrates coalescing erythematous papules into larger plaques on the bilateral forearms.

**Fig 5:**
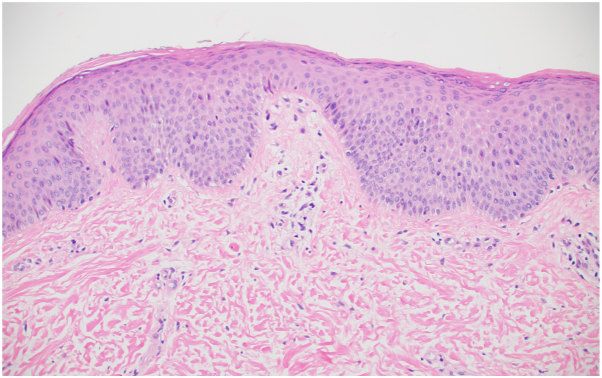
Histopathologic digital slides and descriptions from a punch biopsy of the left wrist. The slide shows subtle psoriasiform and spongiotic dermatitis, with basal layer vacuolization and rare necrotic keratinocytes. (Hematoxylin-eosin stain; original magnification: ×200.)

A 17-year-old boy underwent an unrelated donor bone marrow transplant for idiopathic severe aplastic anemia. The donor’s history of atopy is unknown. The patient had no previous history of atopy. GVHD prophylaxis included mycophenolate mofetil from day −3 to +30 and cyclosporine from day −3 with a taper starting at day +180 in the absence of active GVHD. Risk factors for immune dysregulation after HCT include baseline transplant characteristics, use of a 7 of 8 HLA-matched unrelated donor, reduced intensity conditioning, as well as post-HCT complications including exposure to rituximab for pre-emptive treatment of epstein-barr virus viremia, and prior mild, limited oral chronic GVHD that had resolved with following a course of swish and spit dexamethasone. With wean of cyclosporine GVHD prophylaxis at day +180 post-HCT, he had emergence of symptoms concerning for GVHD including an eczematous rash in bilateral antecubital fossae and over scrotum, weight loss, and intractable nausea/vomiting. These resolved with return to therapeutic dosing of cyclosporine. With renal insufficiency, his immune suppression was changed to sirolimus, but within a month, his GVHD symptoms recurred. One-year posttransplant, he experienced pruritic psoriasiform plaques on his wrists and groin that was more consistent with autoimmune psoriasiform dermatitis as opposed to GVHD (Fig 4).^11^ He experienced concurrent chronic diarrhea, malnutrition, and weight loss initially attributed to *Clostridioides difficile* colitis, but ultimately diagnosed with Crohn disease (with autoreactive B cells). Nutritional deficiencies were evaluated as a potential etiology for his dermatitis, but laboratory testing found normal levels of B1, B2, B3, B6, and B12 and folate. His vitamin C was <5 and he was placed on vitamin C supplementation. A skin biopsy showed subtle psoriasiform dermatitis without features of GVHD (Fig 5). Topical triamcinolone 0.1% ointment and tacrolimus 0.1% ointment were initiated, but he continued to have significant skin flares on the scalp and extremities.

At 3 years post-HCT while on infliximab for Crohn disease, a repeat skin biopsy was performed, again demonstrating psoriasiform and spongiotic dermatitis. Infliximab was switched to ustekinumab for any possible tumor necrosis factor-induced psoriasis component that failed to provide any relief after several months. Because of inadequate clearance with psoriasis biologics, dupilumab was trialed. He was started on a loading dose of 400 mg followed by 200 mg every 14 days. He continued dupilumab 200 mg every 3 weeks. After 2 months of dupilumab treatment the patient’s skin showed near complete clearance. He has continued using dupilumab for a total of 3.5 years and is tolerating well without side effects. He has not yet tried to wean off the dupilumab. Interestingly, multiple biopsies revealed no GVHD, and the patient had no response to traditional GVHD therapies. The finding of concurrent Crohn disease and identification of autoreactive B cells suggests his dermatitis to be autoimmune, consistent with prior reports.^11^

## Discussion

Eczematous GVHD after HCT has many presentations, including lichen-planus-like, morphea-like, psoriasiform, dermatomyositis-like, and lupus-like. The differential includes de novo eczematous dermatitis, possible transfer of atopy from donor to host, and eczematous GVHD. More recently, one type of eczematous dermatitis, known as AD-like GVHD has been described. It was initially in the context of chronic GVHD, but more recently also in the context of acute GVHD.^3^ It is unclear what fraction of acute and chronic GVHD present as AD-like GVHD or other eczematous eruptions. The pathogenesis of eczematous dermatitis after HCT remains poorly understood, making treatment challenging.

Eczematous dermatitis post-HCT is currently treated with conventional immunotherapies, steroids, and phototherapy. However, the long-term effects of chronic immunosuppression and steroid use are undesirable in pediatric patients and alternative therapies are needed. Research suggests that in AD-like GVHD there is immune dysregulation with a skewing toward type 2 inflammation, as evidenced by elevated levels of IgE, eosinophils and T-helper 2 cells; this is akin what is seen in AD.^6^^,^^12^ The prevalent T-helper 2 profile highlights the role for dupilumab, a monoclonal antibody directed against the IL-4 receptor α, leading to disruption of the IL-4 and IL-13 signaling pathways, preferentially downregulating the T-helper 2 pathway.^9^^,^^13^

Our 3 cases highlight that eczematous dermatitis post-HCT can occur in a variety of settings, including acute GVHD, overlap GVHD, and psoriatic-autoimmune reaction, suggesting that many subtypes of eczematous dermatitis post-HCT share enough physiology to respond well to dupilumab (Table I).^1^^,^^2^^,^^14^ Despite the varied reasons for HCT in our patients, all 3 patients achieved near complete clearance of their eczematous reaction, had decreased pruritus, and improved quality of life using dupilumab. Furthermore, all 3 of these patients’ eczematous reactions responded to dupilumab despite being refractory to other systemic topical therapies.

**Table I:** List of all cases of eczematous dermatitis post-HCT that are treated with dupilumab

Manuscript	Age in years (at dupilumab treatment)	Sex	Primary disease	Conditioning regimen	Risk factors for GVHD	Onset of post-HCT AD-dermatitis (setting)	GVHD prophylaxis	Systemic dermatitis therapies	Topical corticosteroids	Topical tacrolimus	Photo therapy	History of atopy	Dupilumab	Duration of follow-up from dupilumab (y)	Response
Case 1	15	M	Hyper-IgE syndrome	MAC: busulfan, fludarabine	Underlying disorder of immune regulation, h/o AD, MAC	3 mo (acute GVHD)	PTCy, MMF, tacrolimus	Tacrolimus, sirolimus, prednisone	+	+	−	Yes	400 mg loading dose 200 mg SC q14d Maintenance dose	0.75	GVHD resolved. Complete clearance and still clear after 9 mo. Off dupilumab and other systemic therapies.
Case 2	19	M	AML	MAC: fludarabine, busulfan, thiotepa, rATG	Mismatched donor, MAC	5 mo (overlap GVHD)	Tacrolimus, MMF	Sirolimus, prednisone, ruxolitinib, rituximab	+	+	−	No	300 mg SC q14d Maintenance dose (no loading dose)	0.17	Improved pruritus and erythema after 2 mo. Patient deceased due to *Aspergillus* pneumonia.
Case 3	17	M	Idiopathic SAA	RIC: fludarabine, CyC, low dose TBI, rATG	Mismatched unrelated donor, rituximab use to treat EBV viremia, prior mild, limited oral cGVHD	12 mo (autoimmune disease)	Tacrolimus, MMF	Infliximab, ustekinumab	+	+	+	No	400 mg loading dose 200 mg SC q21d Maintenance dose	3.5	Eczematous reactions completely resolved. Remains on dupilumab, off all other systemic therapies.
Larijani et al^2^	6	M	Hurler syndrome	Not reported	Unrelated donor	1 mo (acute GVHD)	Not reported	Prednisone, tacrolimus, ruxolitinib	+	−	Unknown	No	400 mg loading dose 200 mg SC q28d Maintenance dose	1.5	GVHD resolved. Remains on dupilumab, off all systemic therapy.
Larijani et al^2^	18	F	Hypodiploid pre-B ALL	Not reported	PBSCT	3 mo (chronic GVHD)	Not reported	Tacrolimus, MMF	+	+	Unknown	No	400 mg loading dose 200 mg SC q28d Maintenance dose	2	GVHD resolved. Remains on dupilumab, off all systemic therapy.
Larijani et al^2^	5	F	SCID	Not reported	Unrelated donor	5 mo (chronic GVHD)	Not reported	Tacrolimus, MMF, ruxolitinib	+	+	Unknown	No	200 mg SC q14d Maintenance dose (no loading dose)	2	GVHD resolved. Remains on dupilumab, off all systemic therapy.
Larijani et al^2^	9	F	Hurler syndrome	Not reported	Unrelated donor	1 mo (acute GVHD)	Not reported	Tacrolimus, MMF	+	+	Unknown	No	600 mg loading dose 300 mg SC q21d Maintenance dose	0.67	Unresponsive. Mild GVHD remains. Switched to ruxolitinib.
Belmesk et al^1^	3	M	PNP SCID	RTC MAC: busulfan, fludarabine, alemtuzumab	Unrelated donor, RTC MAC	6 mo (chronic GVHD)	CsA, MMF	Prednisone, rituximab, MMF, sirolimus, ruxolitinib	+	+	+	No	300 mg SC q14d maintenance dose Loading dose unclear	0.25	GVHD resolved, remains on dupilumab and sirolimus
Belmesk et al^1^	8	M	Fanconi anemia	Fludarabine, CyC	Underlying disorder of DNA repair defect	6 mo (chronic GVHD)	ATG, CsA, MMF	Prednisone, sirolimus	+	+	+	No	200 mg SC q14d Maintenance dose (no loading dose)	0.17	Mild GVHD remains, remains on dupilumab
Belmesk et al^1^	2	M	Wiskott-Aldrich syndrome	RTC MAC: busulfan, fludarabine, alemtuzumab	Underlying immune deficiency with associated rash, RTC MAC	1 mo (acute GVHD)	CsA, MMF	Prednisone, CsA, MMF, tacrolimus, sirolimus, ruxolitinib, rituximab	+	+	+	Yes	300 mg SC q28d Maintenance dose (no loading dose)	1	GVHD resolved. Off dupilumab and all other systemic therapies.
Belmesk et al^1^	5	M	Idiopathic SAA	RIC: fludarabine, alemtuzumab	Not reported	1 mo (acute GVHD)	CsA	CsA	+	+	−	No	300 mg SC q28d Maintenance dose (no loading dose)	0.17	Mild GVHD remains, on CsA and dupilumab.
Tierney et al^14^	2	M	Hurler syndrome	Not reported	Unrelated donor	2 mo (acute GVHD)	Not reported	Methylprednisolone, CsA, ruxolitinib, ECP	+	+	+	No	300 mg SC q14d (no loading dose) Maintenance dose (no loading dose)	1.5	Mild GVHD remains, on dupilumab, off all other systemic therapies.

*AML*, Acute myeloid leukemia; *ATG*, anti-thymocyte globulin; *cGVHD*, chronic graft-versus-host disease; *CsA*, cyclosporine; *CyC*, cylophosphamide; *EBV*, epstein-barr virus; *ECP*, extracorporeal photochemotherapy; *F*, female; *GVHD*, graft-versus-host disease; *h/o*, history of; *HCT*, hematopoietic cell transplant; *M*, male; *MAC*, myeloablative conditioning; *MMF*, mycophenolate mofetil; *PBSCT*, peripheral blood stem cell transplant; *PNP SCID*, purine nucleoside phosphorylase deficiency SCID; *pre-B ALL*, pre-B acute lymphocyte leukemia; *PTCy*, post-transplant cyclophosphamide; *q14d*, every 14 days; *q21d*, every 21 days; *q28d*, every 28 days; *rATG*, rabbit anti-thymocyte globulin; *RIC*, reduced intensity conditioning; *RTC*, radiation with chemotherapy; *SAA*, severe aplastic anemia; *SC*, subcutaneous*SCID*, severe combined immunodeficiency; *TBI*, total body irradiation.

One of our cases is rare in that most patients do not undergo HCT for Hyper-IgE syndrome; this is the first time that dupilumab use has been shown to be effective both pre-HCT and post-HCT for symptoms of AD in this setting.^15^ Interestingly, one patient only required dupilumab for 7 months to resolve his eczematous dermatitis, whereas another requires ongoing treatment years later, suggesting that there may be differences in duration of dupilumab needed for eczematous reactions post-HCT. Further investigation is needed to delineate the differential response to dupilumab in eczematous dermatitis post-HCT. Furthermore, it is unclear how eczematous GVHD reactions differ from eczematous dermatitis in transplant-naïve individuals and how this physiology impacts dupilumab efficacy.

Our cases, and others, support the notion of treating patients with “like” dermatoses similarly to their transplant-naïve counterparts, hypothesizing that similarities in pathology would translate to similar responses. Further investigations are needed to determine the appropriate placement of dupilumab in therapy for in eczematous GVHD. To our knowledge, there are no clinical trials employing dupilumab or other biologics targeting type 2 inflammation to treat any cutaneous form of GVHD. Future clinical trials using dupilumab as first-line systemic therapy in cutaneous eczematous GVHD post-HCT are warranted.

## Conflicts of interest

Dr Maguiness is a cofounder of Stryke Club, personal care for teenage boys and has participated on an advisory board for Regeneron Pharmaceuticals. Dr Ebens is a consultant for Chiesi USA (speaking engagement on therapies in recessive dystrophic epidermolysis bullosa), Ensoma Inc (clinical trial development for chronic granulomatous disease gene therapy), Maro Bio (clinical trial development for a novel nongenotoxic conditioning approach), and serves on the data safety monitoring committee for a trial of telomere elongation sponsored by Elixirgen Therapeutics. Dr Boull is a consultant for SpringWorks Therapeutics (consensus guidelines on treatment for cutaneous toxicities to MEKi). Drs Dick, Truong, and Miller have no conflicts of interest to declare.
